# *In vitro* anti- biofilm and anti-bacterial activity of *Sesbania grandiflora* extract against *Staphylococcus aureus*

**DOI:** 10.1016/j.bbrep.2017.10.004

**Published:** 2017-10-23

**Authors:** Arumugam Dhanesh Gandhi, Dhandapani Kayal Vizhi, Kubendiran Lavanya, V.N. Kalpana, V. Devi Rajeswari, Ranganathan Babujanarthanam

**Affiliations:** a Department of Biotechnology, Thiruvalluvar University, Serkkadu, Vellore 632115, Tamil Nadu, India; b Department of Biomedical Sciences, School of Biosciences and Technology, VIT University, Vellore 632014, Tamil Nadu, India

**Keywords:** *Sesbania grandiflora*, Extra polymorphic substances (EPS), FTIR, *Staphylococcus aureus*, Congo-red assay

## Abstract

The main objective of this research is to investigate the anti-biofilm and anti-bacterial activity of *Sesbania grandiflora* (*S. grandiflora) against Staphylococcus aureus. S. grandiflora* extract were prepared and analyzed with UV –Vis spectroscopy, Fourier transform infrared spectroscopy, Dynamic light scattering. Biofilm forming pathogens were identified by congo-red assay. Quantification of Extracellular polymeric substance (EPS) particularly protein and carbohydrate were calculated. The efficacy of the herbal extract *S. grandiflora* and its inhibition against the pathogenic strain of *S. aureus* was also evaluated. The gradual decrease or disappearance of peaks reveals the reduction of protein and carbohydrate content in the EPS of *S. aureus* when treated with *S. grandiflora*. The antibacterial activity of *S. grandiflora* extract against the bacterial strain *S. aureus* showed that the extract were more active against the strain. To conclude, anti-biofilm and antibacterial efficacy of *S. grandiflora* plays a vital role over biofilm producing pathogens and act as a good source for controlling the microbial population.

## Highlights

• Quantification of EPS particularly protein and carbohydrate were calculated.
• Efficacy of the S.grandiflora and its inhibition against the Staphylococcus aureus was evaluated.
• *S. grandiflora* acts as a good source for controlling the microbial population.
• It can be used as natural preservative ingredients in food and/or pharmaceuticals.

## 1 Introduction

Bio films are defined as microbial communities of cells attached either to a biotic or abiotic surface enclosed in a complex EPS (Extra cellular Polymeric Substance). Biofilms are formed with interaction among microbial aggregates, filamentous bacterial strains, organic and inorganic particles, which are held together by EPS [1], [2]. EPS majorly comprised of four substance called carbohydrate, protein, lipids and extracellular DNA (e DNA). The biofilm acts as a protective barrier and provides resistance against antibiotics, degrading enzymes, protozoan grazers and host immune response [3], [4]. Some bacterial biofilms have been reported to have useful effects on sewage treatment plants, food chains etc [5]. Moreover, biofilms also posses diverse problems in food industry, medicine, biofouling of boats, cooling towers, water pipes etc. The regular use of antibiotics makes the biofilm producing bacteria resistant to antimicrobial agents [6]. Hence, the development of anti-biofilm strategies is therefore a major interest and currently constitutes an important field of investigation in which environmentally friendly anti-biofilm molecules or organisms are highly valuable.

Traditionally, humans have utilized crude extracts of medicinal plants as curative agents for various ailments. Plant extracts and other biologically active compounds isolated from leaves, stems, and roots have gained interest in anti-biofilm activity [7], [8], [9]. Taking this as an initiative, *S. grandiflora* were selected to investigate the anti-biofilm and anti-bacterial activity.

*S. grandiflora* (Family: Fabaceae) is a small, loosely branching tree. It is used as an important dietary nutritive source. The leaves are traditionally used to treat nasal catarrah, nyctalopia and cephalagia. The literature review reveals that, *S. grandiflora* possess antioxidant, antiuroithiatic, anticonvulsive, anti-arthritic, anti-infammatory, anti-helminthic, anti-bacterial and anxiolytic activity [10], [11], [12].

The aim of this study was to investigate the anti-biofilm and antibacterial activity of *S. grandiflora against S. aureus.*

## 2 Materials and methods

### 2.1 Plant material

**Fig. 1:**
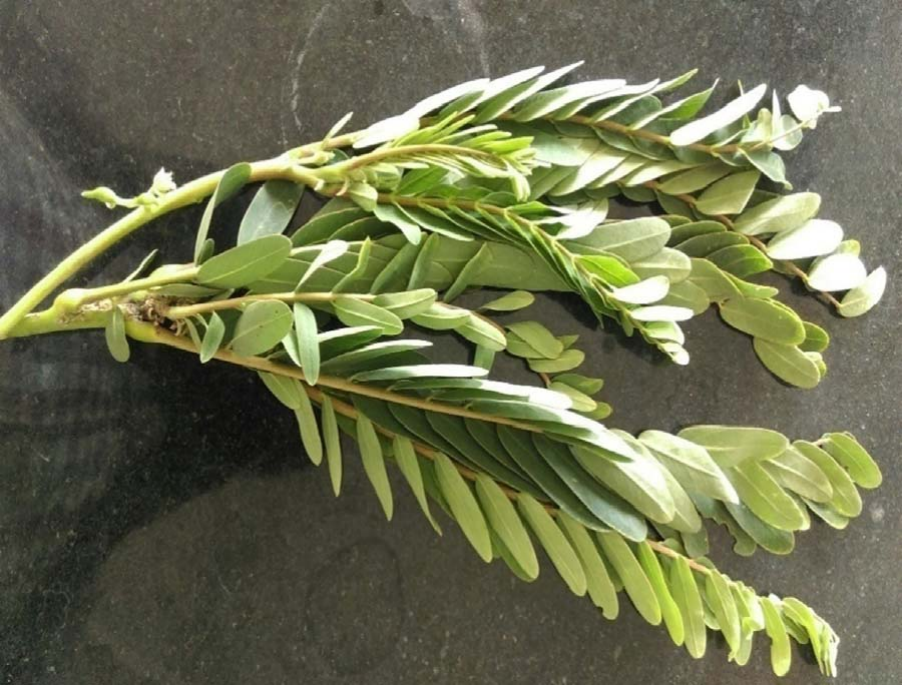
Sesbania grandiflora.

The leaves of *S. grandiflora* were collected from Vellore district. The collected leaves were washed with double distilled water and were air-dried at room temperature for two weeks and coarsely powdered [13] (Fig. 1).

### 2.2 Preparation of S. grandiflora seed extract

**Fig. 2:**
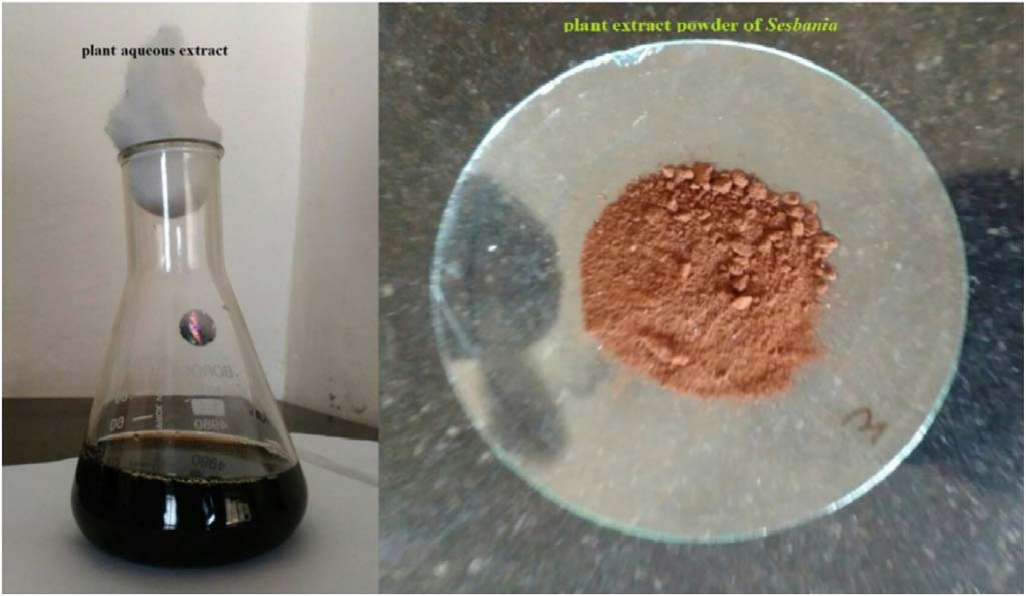
Aqueous extract of *Sesbania grandiflora*.

The powdered sample was boiled with 100 ml of sterile distilled water at 60 °C for 1 h. Then, the extract was filtered through Whatman No. 1 filter paper and used for further experiments (Fig. 2).

### 2.3 Antimicrobial activity

The antimicrobial activities of the leaves extract was determined by disc diffusion method. Nutrient agar (NA) plates were prepared, sterilized and solidified. The culture *Staphylococcus aureus* was swabbed uniformly onto the NA plates using sterile cotton swabs. Plates were incubated at 37 °C for 24 h [11], [14], [15].

### 2.4 Biofilm formation in Congo red agar

NA plates were prepared, inoculated and incubated at 37 °C for 24 h. The cultured plate was flooded with 0.2% congo red dye. After 15 min, the plates were destained with 1 M NaCl [16].

### 2.5 EPS production

*Staphylococcus aureus* culture of 20 µl (222 CFU) was added in freshly prepared 50 ml of sterile nutrient broth and were treated with *Sesbania grandiflora* plant extract powder of 3 different concentration (250, 500,750 mg) and one is maintained as control without the addition of plant extract then the samples were incubated at 37 °C for 24 h at 140 rpm. Control and the treated samples were centrifuged at 12,000 rpm for 15 min at 4 °C and to the supernatants were collected, to that three volumes of ice cold isopropanol was added and incubated overnight at −25 °C. The incubated samples were centrifuged at 5000 rpm for 30 min and pellets were collected (EPS) and air dried at room temperature. The collected EPS of control and treated were purified, and stored for further analysis [17].

### 2.6 EPS characterization

The control and treated EPS samples were characterized by UV – Visible Spectroscopy analysis, Fourier Transform Infrared Spectroscopy (FTIR), Light Scattering analysis [18], [19].

### 2.7 Emulsifying activity

The control and treated EPS solution (2 mg/ml) was heated at 100 °C for 15 min followed by cooling (25 °C) and the volume was made upto 2 ml using phosphate-buffered saline. 1 ml of olive oil was added, vortexed for 1 min and the absorbance was measured after 30 min at 540 nm. The emulsifying activity was calculated in terms of percentage decline in the absorbance at different time intervals [19].

### 2.8 Quantification and inhibition of EPS components

EPS was quantified by measuring proteins (Lowry's method) and carbohydrates (Anthrone method) [17].

The percentage of EPS inhibition was calculated using the formula

%inhibition=controlOD–TreatedOD/ControlOD×100

## 3 Results

### 3.1 Antimicrobial activity

The anti microbial potential of *S. grandiflora* was evaluated according to their zone of inhibition against *S. aureus* pathogens and the results were compared with the activity of the standards viz, Ampicillin. 40 µl of *S. grandiflora* were found to be more effective against *S. aureus* when compared with the standards

### 3.2 Biofilm formation in Congo red agar

**Fig. 3:**
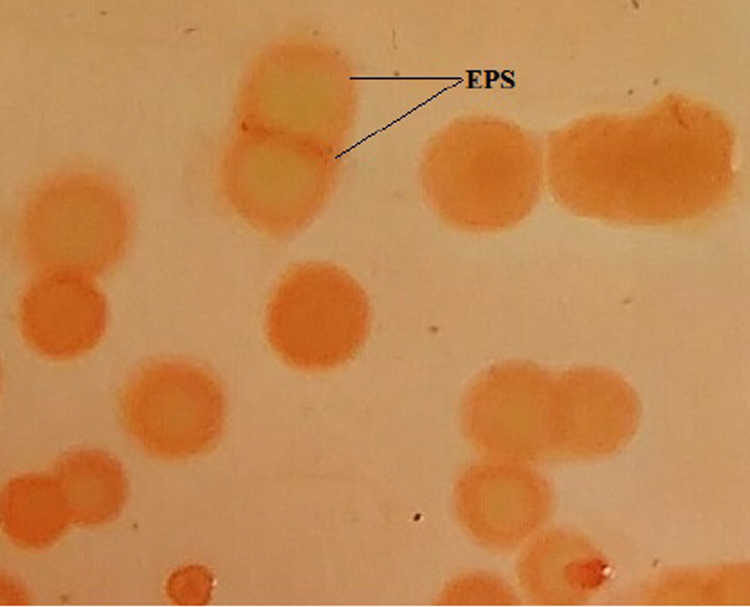
Congo red assay.

Biofilm formation of *S. aureus* on NA plates supplemented with congo red appeared as dark red around the bacteria colonies. The biofilm produced by bacteria is made up of exopolysaccharide matrix that protects microbes from host immune system and anti-microbial therapy. Based on our results, *S. aureus* displayed highest biofilm forming ability (Fig. 3).

### 3.3 EPS production

Maximum EPS was produced after 48 h in Nutrient broth by *S. aureus.*

### 3.4 EPS characterization

#### 3.4.1 UV visible spectroscopy

The UV-Vis analysis was used for the identification of optical property of *S. grandiflora*.

**Fig. 4:**
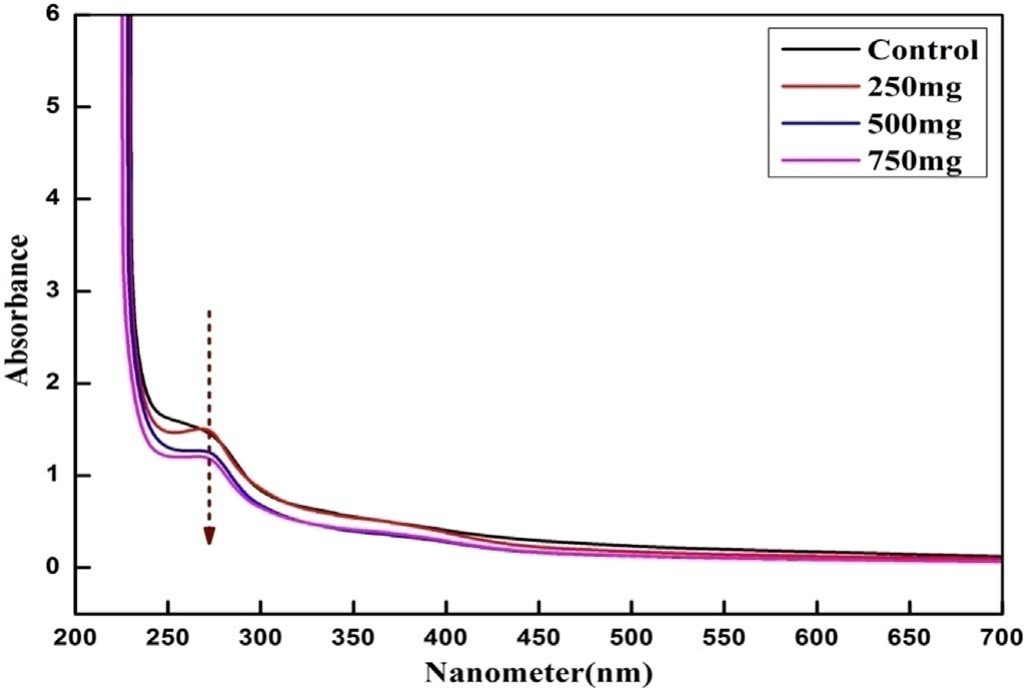
UV-Visible spectrum of EPS.

The high exciton binding energy was responded at 270 nm as clear absorption band (Fig. 4).

#### 3.4.2 FTIR

**Fig. 5:**
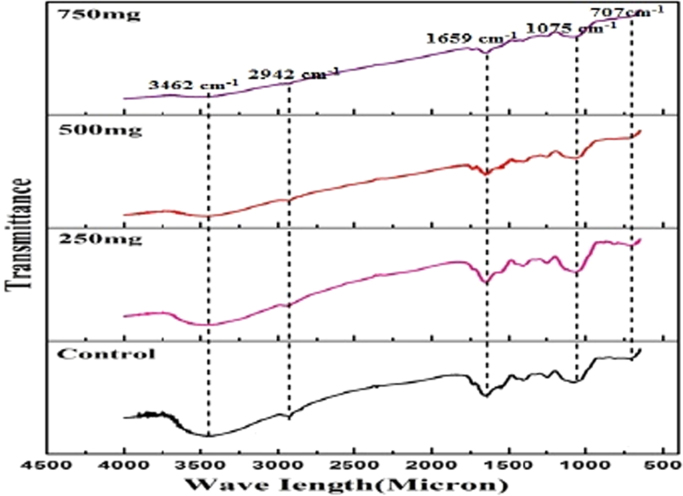
FTIR spectrum of EPS.

Fourier Transform Infrared spectroscopy was used to find out the biomolecules. FTIR spectrum shows peak at 707 cm^−1^, 1075 cm^−1^, 1659 cm^−1^, 2942 cm^−1^, 3462 cm^−1^ which confirms the presence of carboxylic group, O-acetyl ester linkage bond, C- H stretching of sugar molecules, C-H functional groups and hydroxyl group respectively. The gradual decrease or disappearance of peaks reveals the reduction of protein and carbohydrate content in the EPS of *S. aureus* when treated with *S. grandiflora* (Fig. 5).

#### 3.4.3 Dynamic light scattering

**Fig. 6:**
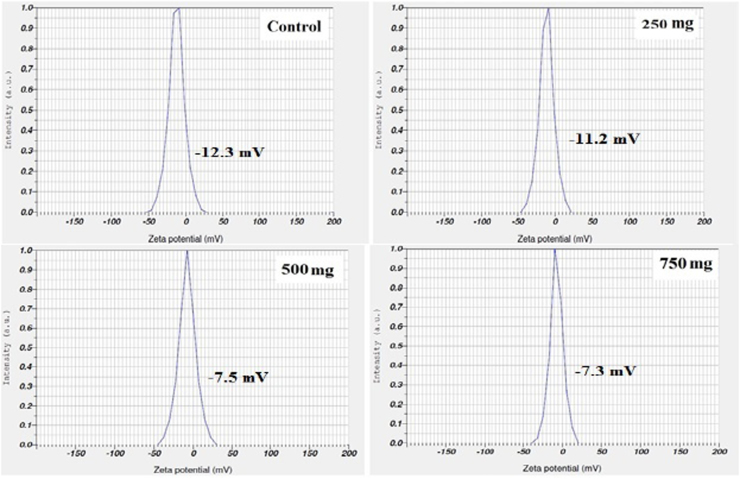
Dynamic light scattering of EPS.

The stability of EPS was analyzed by Zeta potential which is high in the control when compare to treated EPS. The value number of the zeta potential showed the stability of the EPS. This analysis is mainly used to check the cell wall penetration of EPS and stability of the bacteria (Fig. 6; Table 1).

**Table 1:** Zeta potential peaks and its determination.

**EPS sample**	**Stability**
**C**	−12.3 mV
**T1**	−11.2 mV
**T2**	−7.5 mV
**T3**	−7.3 mV

### 3.5 Emulsifying activity

**Fig. 7:**
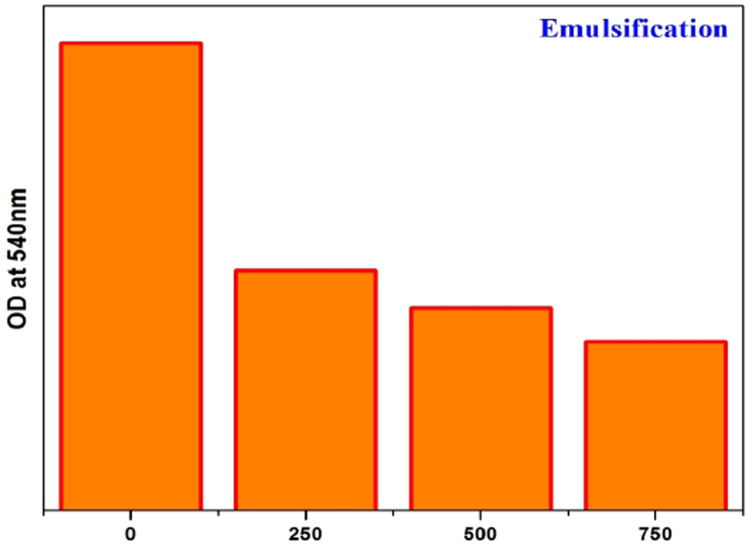
Histogram of emulsification index.

A stable emulsion of the EPS was observed with Olive oil. This yielded 48.51%, 53.62%, 72.11% (at different concentration 250, 500, 750 mg/ml) emulsifying activity with olive oil after 30 min of incubation. The emulsifying activity of EPS was measured before 30 min because emulsification will break within 30 min, due to the stability of the sample. The presence of both hydrophilic (hydroxyl) and hydrophobic (aliphatic CH_2_) functional groups may be responsible for its emulsifying property (Fig. 7).

### 3.6 Quantification and inhibition of EPS components

**Fig. 8:**
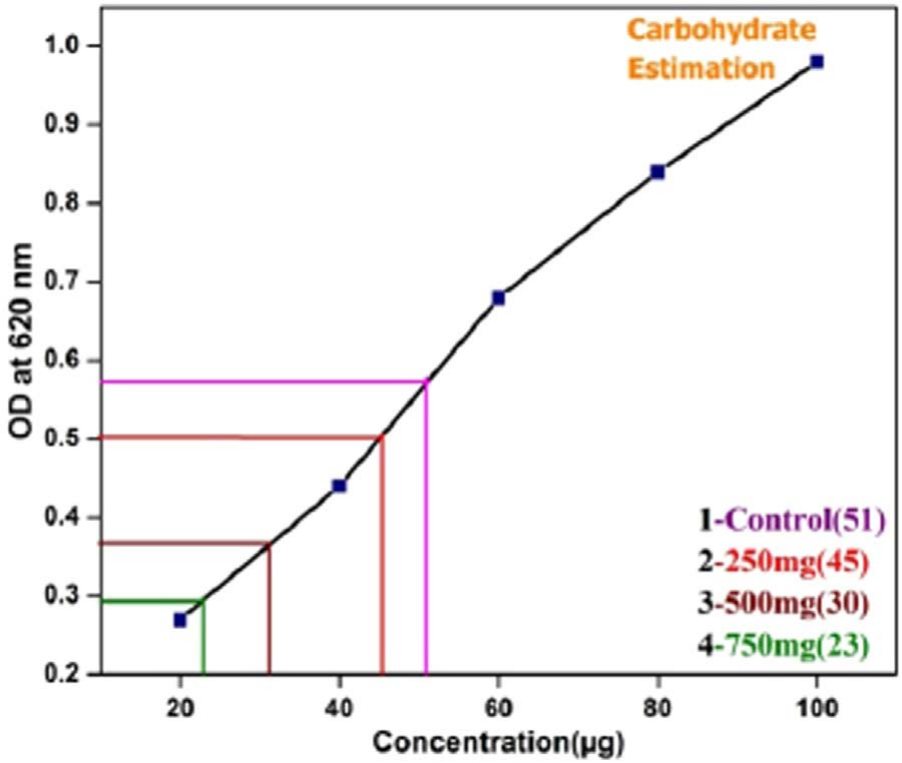
Carbohydrate estimation in EPS.

The optical density of the control and treated EPS revels that the amount and inhibition percentage of the protein in the given sample by measuring at 660 nm and inhibition percentage of carbohydrate in the given sample was measured at 620 nm (Fig. 8; Table 2).

**Table 2:** Comparison of carbohydrate concentration with control.

**S.No**	Concentration of *S. grandiflora* leaf extract (mg)	Carbohydrate concentration present in control and treated EPS (µg)	Percentage of Carbohydrate inhibition
**C**	–	51	–
**T1**	250	45	11.2%
**T2**	500	30	36.71%
**T3**	750	22	49.36%

**Fig. 9:**
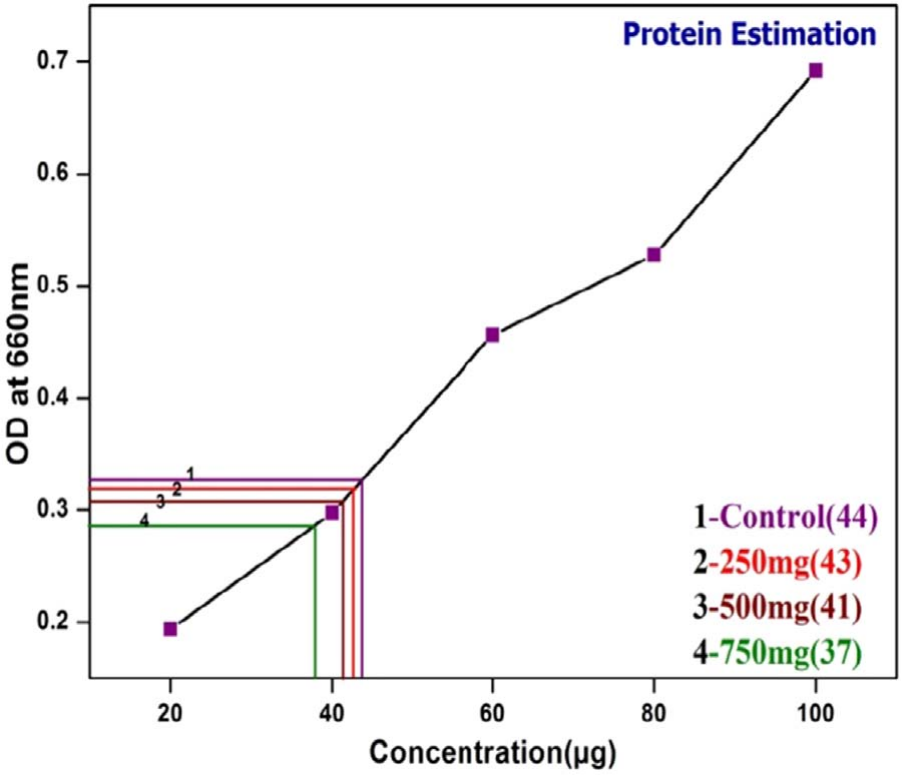
Estimation of Protein in EPS.

The gradual decrease in the amount of protein and carbohydrate in control and treated EPS were calculated in terms of percentage. The amount of protein in EPS decreases with the increase of plant extract. This variation in control and treated EPS is due to increase in plant extract concentration (Fig. 9; Table 3).

**Table 3:** Comparison of protein concentration with control.

**S.No**	**Concentration of*****S. grandiflora*****leaf extract (mg)**	**Protein concentration present in control and treated EPS(µg)**	**Percentage of protein inhibition**
**C**	–	44	–
**T1**	250	41	2.1%
**T2**	500	37	6.5%
**T3**	750	31	14.44%

## 4 Discussion

*S. grandiflora* was extensively studied by different scientists and academicians for its phytopharmacological potentials especially on leaves, flowers and seeds. Recent investigation demonstrated that use of leaf extract of *S. grandiflora* act as a reducing agent and synthesized spherical shaped silver nanoparticles [20]. Moreover, the *S. grandiflora* flower acts as a promising material to develop the active ingredient of anti-plaque toothpaste as well as mouthwash solution [21]. It has been reported that a biofilm is strongly associated with the drug resistance property [22]. Hence, eradication of biofilm is often considered to be a difficult task and therefore use of plant products to inhibit biofilm may be a viable alternative [23].

In this study, the biofilm inhibition potential of *S. grandiflora* against *S. aureus* was evaluated by using quantitative spectroscopic techniques. 40 µg of *S. grandiflora* leaf extract showed potential antibacterial activity against *S. aureus. S. grandiflora* extract have been known to contain alkaloids, flavonoids, saponins, tannins and steroids [24]. These active compounds have the potential to inhibit adhesions and prevent matrix formation. There were reports which state that biofilms acquire resistances to inhibitors under nutrient limited or depleted conditions in contrast to their susceptibility conditions. *S. grandiflora* leaf extract shown to have anti-biofilm efficacy under both nutrient repleted and nutrient depleted conditions, which indicate the presence of bioactive agent in the leaf extract [25].

Carbohydrate and proteins were considered as a main constituent of EPS in pure culture. In this study, the ratio of carbohydrate and protein of the pure culture was comparatively less after treatment with *S. grandiflora* leaf extract [26].

UV- Vis spectroscopy analysis of control and treated EPS revealed a major absorption peak at 270 nm, which revealed that the intensity of the peak is directly proportional to the concentration of the EPS. This was similar to surface Plasmon vibration with the previous work. FTIR results represented that the active biomolecules present in the leaves of *S. grandiflora* was responsible to inhibit the biofilm formation [27]. The broad stretching of 3462 cm^−1^ indicates the presence of hydroxyl group. The band at 2942 cm^−1^ was due to the presence of C-H functional groups, which revealed the presence of sugar. Another peak at 1659 cm^−1^ could attribute to the C-H stretching of mannose or galactose [28]. The absorption peak at 1075 cm^−1^ may be attributed to O-acetyl ester linkage bond. DLS showed that the decrease in volume number indicate the degradation of the protein molecule in EPS, and in-turn revealed the decreased stability than the Control [29].

## 5 Conclusion

In this study, a suitable and efficient method for the EPS extraction from *S. grandiflora* biofilms was achieved. This study also suggests that *S. grandiflora* extract possesses compounds with potential antimicrobial properties. To conclude, the aqueous extract of *S. grandiflora* leaf extract showed evidence of high anti-biofilm and anti-bacterial activity property against *S. aureus*. Further research is needed to elucidate the antimicrobial agents from *S. grandiflora* that are pharmacologically important using advance techniques in an eco-friendly approach
