# Supplementary material for: In vitro anti- biofilm and anti-bacterial activity of Sesbania grandiflora extract against Staphylococcus aureus
Source: Biochem Biophys Rep. 2017 Oct 23;12:193–7. doi: 10.1016/j.bbrep.2017.10.004 (PMC5655386; doi:10.1016/j.bbrep.2017.10.004)
Supplement: Supplementary file 1 — Supplementary material [file mmc1.docx]

**Conflict of Interest statement**

**Manuscript title**

“*In vitro* anti- biofilm and anti-bacterial activity of *Sesbania grandiflora* extract against *Staphylococcus aureus”*

**Authors :**

Arumugam Dhanesh Gandhi^a^, Dhandapani Kayal Vizhi^a^, Kubendiran Lavanya^a^, V. N. Kalpana^b^, Devi Rajeswari V.^b*^, Ranganathan Babujanarthanam^a*^

**Authors Affiliation**

*^a^Department of Biotechnology, Thiruvalluvar University, Serkkadu, Vellore–632 11, Tamil Nadu, India.*

*^b^Department of Biomedical Sciences, School of Biosciences and Technology, VIT university, Vellore, Tamil nadu, India.*

***Corresponding author:**

*^1^Ranganathan Babujanarthanam, Department of Biotechnology, Thiruvalluvar University, Serkkadu, Vellore–632 11, Tamil Nadu, India.* [*babukmg@gmail.com*](mailto:babukmg@gmail.com)

*^2^V. Devi Rajeswari, Department of Biomedical Sciences, School of Biosciences and Technology, VIT University, Vellore-632014, Tamil Nadu, India.* [*sdevirajeswari@gmail.com*](mailto:sdevirajeswari@gmail.com)

The authors declare that there is no Conflict of interest between them . I warrant that the article is the Authors' original work. I warrant that the article has not received prior publication and is not under consideration for publication elsewhere. On behalf of all Co-Authors, the corresponding Author shall bear full responsibility for the submission.

I Dr. V. Devi Rajeswari Submitting this on behalf of all authors.

Sincerely,

Dr. V. Devi Rajeswari
